# Bridging minds and machines in Industry 5.0: neurobiological approach

**DOI:** 10.3389/fnhum.2024.1427512

**Published:** 2024-08-27

**Authors:** Valentina Rueda-Castro, Jose Daniel Azofeifa, Julian Chacon, Patricia Caratozzolo

**Affiliations:** ^1^Institute for the Future of Education, Tecnologico de Monterrey, Monterrey, Mexico; ^2^School of Engineering and Sciences, Tecnologico de Monterrey, Mexico City, Mexico

**Keywords:** EEG, Industry 5.0, human-centered, neuropsychology, biometric assessment, abilities, neuroeducation

## Abstract

**Introduction:**

In transitioning from Industry 4.0 to the forthcoming Industry 5.0, this research explores the fusion of the humanistic view and technological developments to redefine Continuing Engineering Education (CEE). Industry 5.0 introduces concepts like biomanufacturing and human-centricity, embodying the integration of sustainability and resiliency principles in CEE, thereby shaping the upskilling and reskilling initiatives for the future workforce. The interaction of sophisticated concepts such as Human-Machine Interface and Brain-Computer Interface (BCI) forms a conceptual bridge toward the approaching Fifth Industrial Revolution, allowing one to understand human beings and the impact of their biological development across diverse and changing workplace settings.

**Methods:**

Our research is based on recent studies into Knowledge, Skills, and Abilities taxonomies, linking these elements with dynamic labor market profiles. This work intends to integrate a biometric perspective to conceptualize and describe how cognitive abilities could be represented by linking a Neuropsychological test and a biometric assessment. We administered the brief Neuropsychological Battery in Spanish (Neuropsi Breve). At the same time, 15 engineering students used the Emotiv insight device that allowed the EEG recollection to measure performance metrics such as attention, stress, engagement, and excitement.

**Results:**

The findings of this research illustrate a methodology that allowed the first approach to the cognitive abilities of engineering students to be from neurobiological and behavioral perspectives. Additionally, two profiles were extracted from the results. The first illustrates the Neuropsi test areas, its most common mistakes, and its performance ratings regarding the students' sample. The second profile shows the interaction between the EEG and Neuropsi test, showing engineering students' cognitive and emotional states based on biometric levels.

**Discussions:**

The study demonstrates the potential of integrating neurobiological assessment into engineering education, highlighting a significant advancement in addressing the skills requirements of Industry 5.0. The results suggest that obtaining a comprehensive understanding of students' cognitive abilities is possible, and educational interventions can be adapted by combining neuropsychological approaches with EEG data collection. In the future, it is essential to refine these evaluation methods further and explore their applicability in different engineering disciplines. Additionally, it is necessary to investigate the long-term impact of these methods on workforce preparation and performance.

## 1 Introduction

The transition from Industry 4.0 to Industry 5.0 has significant implications for Higher Education Institutions (HEIs) as they play a critical role in preparing the workforce for the changing industrial landscape (Gürdür Broo et al., 2022). Incorporating cutting-edge technologies into academic programs is a key focus to ensure that graduates can efficiently collaborate with intelligent machines (Maddikunta et al., 2022). However, it is equally important for HEIs to emphasize lifelong learning and professional development to support individuals in navigating the demands of the digital economy and equipping them with the necessary skills for the future workforce. Within the context of Industry 4.0 and 5.0, it must also be considered that employees who are invited to train, adopt, and apply innovative combinations of methodologies with emerging technologies may have concerns regarding the effectiveness and applicability of these new approaches, therefore seeking to understand these perceptions is essential to adapt training programs effectively and ensure smooth integration of new technologies into the workplace. The insights from these employees can help design more inclusive and responsive training initiatives that address their concerns and improve their willingness to engage with new technologies, thereby facilitating the transition to Industry 5.0 (Mystakidis et al., 2021; Kazakou and Koutromanos, 2024).

The human-centric nature of Industry 5.0 highlights the need to identify and assess the Skills and Abilities (S&A) of engineering graduates (SkillsFuture Singapore, 2021; Future-Skills, 2023). Effective human-machine collaboration requires individuals to be matched with tasks and roles that capitalize on their strengths, expertise, and creativity. Assessing the S&A of engineering graduates ensures that they possess the competencies required to make informed decisions and navigate the development and deployment of technology. Given the rapid technological advancements in Industry 5.0, engineers must continuously update their skill sets to remain relevant in the changing industrial landscape (Caratozzolo et al., 2024; Saniuk and Grabowska, 2024). Unfortunately, Industry 5.0 also contributes to the engineering workforce's skills gap and mismatch problem, where the skills gap refers to a misalignment between the skills employers demand and those possessed by the workforce, posing significant challenges for companies striving to remain competitive (Nayernia et al., 2022). Furthermore, Industry 5.0 requires engineers to have diverse skills beyond traditional frameworks, such as oral and written communication, attention to detail, memory encoding, and memory retrieval. This can lead to mismatches between graduates' skills and the requirements of their job roles (Aggarwal et al., 2022).

To address these challenges and enhance the cognitive capabilities of engineering students, Cognitive Neuroscience (CN) plays a crucial role. CN is an interdisciplinary subject that explores the connections between brain functions and mental processes (Banich and Compton, 2018). It has made significant contributions to understanding cognitive processes such as memory, attention, language, and emotions, as well as the learning process itself (Parong and Mayer, 2021; Xu et al., 2021; Zhang et al., 2021). The advancement of CN has enabled the implementation of biometric methodologies, such as EEG data collection, to gain insights into the neural mechanisms associated with specific S&A. By combining EEG data collection with neuropsychological assessments, researchers can understand and enhance students' cognitive capabilities, including oral and written communication, critical reading, attention to detail, and memory functions (Davidesco et al., 2021). Applying a neuroeducation approach to engineering students can offer meaningful insights into complying with the human-centricity of Industry 5.0. By focusing on understanding and enhancing cognitive and socio-emotional skills essential for effective human-machine collaboration and innovation, educators can tailor instructional strategies and interventions to optimize the learning experience (Bhargava and Ramadas, 2022).

Insights into students' cognitive strengths, learning preferences, and emotional regulation abilities can be gained through EEG data collection and neuropsychological assessments. For this reason, this study intends to evaluate the proficiency levels of engineering students in the relevant skills and abilities for Industry 5.0. This evaluation is based on the correlation between the cognitive profiles obtained from a neuropsychological test and the neurobiological markers collected with the EEG device.

## 2 Overview

This section examines the transition from Industry 4.0 to Industry 5.0, highlighting the important implications for industries and higher education institutions. Explores the human-centric approach inherent to Industry 5.0, emphasizing the importance of identifying and assessing the skills and abilities necessary for effective human-machine collaboration. Additionally, this section presents the role of Cognitive Neuroscience and Neuroeducation in improving the cognitive capabilities of engineering students, providing a foundation for future workforce preparation in an increasingly digitalized and interconnected world.

### 2.1 Industry 5.0 and human centered design

The transition from Industry 4.0 to Industry 5.0 carries significant implications for industries because of changes in operations, driving innovation, efficiency, and flexibility (Xu et al., 2021; Mourtzis et al., 2022). Industry 5.0 also implies challenges and opportunities for society and individuals regarding workforce transformation and ethical considerations (Saniuk et al., 2022). Society must adapt to the changing nature of work, investing in education, infrastructure, and policies that support the transition to Industry 5.0 while experiencing changes in employment patterns, skills requirements, and social dynamics. Individuals must continuously update their skills, embrace lifelong learning, and adapt to evolving job roles and responsibilities in the increasingly digitalized and interconnected world of Industry 5.0 (Zizic et al., 2022; Leon, 2023).

The transition from Industry 4.0 to Industry 5.0 also poses significant implications for Higher Education Institutions (HEIs), which play a critical role in preparing the workforce for the changing industrial landscape (Gürdür Broo et al., 2022; Caratozzolo et al., 2024). Most of the research related to the HEI challenge is related to incorporating cutting-edge technologies, such as Artificial Intelligence, Augmented and Virtual Reality, and Generative Language Models, into academic programs to ensure that graduates can efficiently collaborate with intelligent machines (Maddikunta et al., 2022; Azofeifa et al., 2024). However, HEIs must emphasize lifelong learning and professional development, offering programs and upskilling and reskilling initiatives to support individuals not only in navigating the evolving demands of the digital economy but also in having the requisite skills and competencies for the future workforce (Chin, 2021; Poláková et al., 2023).

One of the main elements of Industry 5.0 is its centrality in human beings (Breque et al., 2021). In various studies, the authors reviewed the relationship between Industry 5.0 and innovation, finding that in the studies carried out so far the concept of human centrality is present in some way, establishing that the advancement of technology must be accompanied by human empowerment, mapping different technologies that can increase resilience, as well as productivity in an industrial context (Akundi et al., 2022; Rowan, 2023; Troisi et al., 2023). Although human centrality is considered a potential enabler in the industry, it is also considered something that can be replaced (Cannavacciuolo et al., 2023).

On the other hand, in the second category, the human aspect is considered necessary for Industry 5.0. It describes it as an “enabling factor of innovation, rather than as a variable that can simply coexist with technology application” (Troisi et al., 2023). Under this category for technology to thrive, its implementation should be based on a human-centric approach. In addition, in the third category, the social impact of Industry 5.0 is being considered in a systems-based approach, where the objective of the innovation processes tends to look for a positive impact on the welfare of human beings (Záklasník and Putnová, 2019), as well as to increase the resilience and sustainability of the system itself (Calp and Bütüner, 2022). Finally, the fourth category delves into the mechanisms related to knowledge management and its relation to an innovation ecosystem as a catalyst for innovation (Carayannis et al., 2022).

Within this context and aims to create a general understanding of human-centricity, Gasson (2003) and Dym et al. (2005) have defined of Human Centered Design as “an approach to design and innovation in which an understanding of potential users drives decision-making” and Van der Bijl-Brouwer and Dorst (2017) description of Human Centered Design as “a group of methods and principles aimed at supporting the design of useful, usable, pleasurable, and meaningful products or services for people.” Given the relevance of Industry 5.0 for companies, it can be useful to understand frameworks such as Human-Centered Design to take the most advantage of daily technological advancements. For effective use of Human Centered Design, it is critical to get an integral understanding of humans (Sanders et al., 2023) in terms of their aspirations (Van der Bijl-Brouwer and Dorst, 2017), desires (Matheson et al., 2015), emotions (Matheson et al., 2015; Van der Bijl-Brouwer and Dorst, 2017), values (Åman et al., 2017), dreams (Sanders et al., 2023), concerns, cultural and political influences (Buchanan, 2001; Zoltowski et al., 2012). Also, it has been seen that the way students experience Human Centered Design can vary depending on the understanding of the context within which it is being used, the way how the students get immersed in the setting of the user, critical experiences, and reflecting on the process itself (Sanders et al., 2023).

The human-centricity of Industry 5.0 necessitates identifying and assessing engineering graduates' Skills and Abilities (S&A) due to its emphasis on placing humans at the forefront of technological innovation and industrial progress (SkillsFuture Singapore, 2021; Future-Skills, 2023). In this paradigm, where human-machine collaboration is central, understanding the capabilities and proficiencies of engineering graduates is crucial for several reasons: firstly, effective human-machine collaboration requires matching individuals with tasks and roles that capitalize on their strengths and expertise, maximizing creativity and innovation (Pizoń and Gola, 2023); secondly, assessing the S&A of engineering graduates ensures that individuals possess the competencies necessary to be more resilient to disruption and make informed decisions throughout the development and deployment of technology (Saniuk and Grabowska, 2024).

Unfortunately, Industry 5.0 also contributes to the engineering workforce's skills gap and mismatch problem: its rapid technological advancements require engineers to update their skill sets to remain relevant continually (Puckett et al., 2020; Brun-Schammé and Rey, 2021). On the one hand, many engineering professionals may struggle to keep up with the rapid pace of change, leading to a gap between the skills demanded by employers and the skills possessed by the workforce (Braun, 2023). On the other hand, Industry 5.0 requires engineers to have diverse skills beyond traditional Knowledge, Skills, and Abilities (KSA) frameworks (Huang et al., 2022; Caratozzolo et al., 2023). For example, some soft skills, such as oral and written communication and critical reading, as well as some Abilities, such as orientation, attention to detail, memory-encoding, and memory-retrieving, are increasingly valued in Industry 5.0 roles. Consequently, recent graduate engineers with narrow specializations may struggle to adapt to the human-centric nature of Industry 5.0 roles, leading to mismatches between their S&A and the requirements of their job roles (Aggarwal et al., 2022; Benitez-Marquez et al., 2022).

The skills gap in the industry refers to a misalignment between the workforce's skills and those that employers demand. This gap poses significant challenges for companies striving to remain competitive in rapidly evolving markets (Schwab, 2017; WEF, 2018; Nayernia et al., 2022). Furthermore, to address the challenges posed by the skills gap, researchers and practitioners have turned to KSA-based taxonomies to categorize and understand essential competencies for various occupations (Caratozzolo et al., 2023). These traditional KSA taxonomies have been critical in shaping educational curricula and informing hiring practices in human resources departments. However, these static taxonomies have struggled to keep pace with the dynamic evolution of the job landscape (Chang et al., 2019; Seemiller and Whitney, 2020).

Some leading occupational organizations have led efforts to develop comprehensive frameworks to understand and address the skills gap, such as the World Economic Forum (WEF, 2021, 2023), NESTA Taxonomy (NESTA, 2023), SkillsFuture of the Government of Singapore (SkillsFuture Singapore, 2021; Fang et al., 2022), the Standard Occupational Classification (SOC) (U. S. Bureau of Labor Statistics, 2018), among others. These frameworks offer insights into employability strategies, reskilling initiatives, and opportunity mapping within the future economy. Through extensive surveys and data analysis, these organizations have identified vital skills and occupations for the future workforce (SkillsFuture Singapore, 2021; WEF, 2021, 2023). Along with this, there are some taxonomy frameworks based on dynamic KSA that are currently being developed, such as the example of the ShapingSkills framework, which, through systematic literature reviews, industrial surveys, and emerging trends in the labor market, together with technical Natural language processing and other current artificial intelligence techniques seek to have the dynamism that the current industry requires regarding the issue of KSA taxonomies that are fresh and functional during the exponential growth of technologies and new requirements within industry 5.0 (ShapingSkills, 2023).

### 2.2 Cognitive neuroscience and neuroeducation

Cognitive Neuroscience (CN) is an interdisciplinary subject that seeks practical and theoretical methodologies that establish connections between brain functions and mental processes (Banich and Compton, 2018). Moreover, CN research has brought relevant biological and psychological discoveries about cognitive processes such as memory (Jimenez et al., 2020; Vaz et al., 2020; Bergmann and Ortiz-Tudela, 2023), attention (Li et al., 2020; Jacob et al., 2021; Niu et al., 2021), language (García et al., 2020; Finlayson et al., 2021; Jiao et al., 2022) and emotions (Alexander et al., 2021; Eslinger et al., 2021; Quadt et al., 2022). Cognitive processes are also involved in the learning process, and recent research is creating learning models to understand the different scenarios where this process occurs (Parong and Mayer, 2021; Zhang et al., 2021; Skulmowski and Xu, 2022).

The advancement of CN has allowed the implementation of biometric methodologies to increase knowledge in other fields such as psychology and education. An example of this is the increasing adoption by researchers of portable EEG measuring devices designed exclusively for research use (Williams et al., 2020). These non-invasive headsets enable data collection in natural environments, such as classrooms, to measure variables that underlie learning like attention (Chen et al., 2017; Sezer et al., 2017; Souza and Naves, 2021). They also use friendly Brain-Computer Interfaces (BCI) made from Machine Learning (ML) and Artificial Intelligence (AI) algorithms.

Recently, it is more common to correlate biometrics such as EEG with different psychological and neuropsychological tests due to the advantages. Tests have evaluated mostly Neuropsychological characteristics such as memory, language, attention, and visuospatial abilities to define executive functions with behavioral results. Nowadays, complementing the observation and definition of these cognitive abilities with biometrical responses makes it possible to create more accurate diagnoses and abilities profiles (Borgianni and Maccioni, 2020). For instance, visuospatial abilities have been correlated with Alpha and Theta brain waves; Jaramillo-Jimenez and his colleagues using the Judgment of Line Orientation test (JLOT) in two groups one with Parkinson's Disease and another group of healthy controls found that when alpha and theta are low in right and left occipital and right parietal areas the performance in visuospatial ability is worse (Jaramillo-Jimenez et al., 2021). On the other hand, models for the prediction of a decline in cognitive domains have also been used in different cognitive tests related to intelligence, visual and verbal memory, and attention supported by multiple neurotechnologies such as MRI Volumetry and Wavelets (Höller et al., 2020). These are examples of how biometrics have been used to complement Neuropsychological assessment. In addition, this combined methodology has been transferred to other fields such as education. This implies that research on cognitive process, previously confined to the field of neuroscience, has incorporated pedagogical methodologies to optimize learning in HE. For instance, a study in 2022 developed a neuroscience-based initiative for teaching skills in HE for the purpose of establishing critical links between neuroeducation and pedagogical theories (Fragkaki et al., 2022). These connections mention Neuroeducation principles such as attention, critical thinking, chucking of content, and emotions along with pedagogical theories such as cognitive load theory, critical pedagogy, zone of proximal development, and meaningful learning, respectively (Fragkaki et al., 2022). Moreover, educational neuroscience directly impacts the professional purpose, patience, and self-confidence of teachers who apply it in classrooms (Ching et al., 2020).

Utilizing neurobiological studies to identify and assess engineering students' S&A offers a range of benefits in understanding and enhancing their cognitive capabilities (Davidesco et al., 2021). Researchers can gain valuable insights into the underlying neural mechanisms associated with specific S&A, such as oral and written communication and critical reading, orientation, attention to detail, memory encoding, and memory retrieval, by employing EEG data collection and neuropsychological assessments (Gkintoni and Dimakos, 2022). These studies provide objective measures of cognitive function, allowing for a more comprehensive evaluation of students' strengths and areas for improvement. Furthermore, neurobiological approaches offer a unique perspective on individual differences in learning styles, information processing, and cognitive strategies, enabling tailored interventions and personalized learning experiences (Bilder and Reise, 2019; Ni et al., 2020; Apicella et al., 2022). By leveraging neurobiological research, educators can develop targeted training programs that optimize student learning outcomes and facilitate successful integration into the workforce for the challenges of Industry 5.0 and beyond.

A neuroeducation approach applied to engineering students can offer meaningful insights into complying with the human-centricity of Industry 5.0 by focusing on understanding and enhancing the cognitive and socio-emotional skills essential for effective human-machine collaboration and innovation (Bhargava and Ramadas, 2022). Educators can gain insights into students' cognitive strengths, learning preferences, and emotional regulation abilities using EEG data collection and neuropsychological assessments (Bilder and Reise, 2019; Ni et al., 2020; Apicella et al., 2022). Furthermore, a neuroeducation approach can help identify and develop the soft skills increasingly valued in Industry 5.0 (Saniuk and Grabowska, 2024).

### 2.3 EEG applications in educational research

Recently, interest in measuring students' brain activity in different learning environments has increased. Portable EEG devices have been particularly useful for this purpose. Various types of these devices have been compared to determine which offers the best signal quality and ease of use (Tsiara et al., 2019), as these instruments are valuable in the field of educational neuroscience. These devices have contributed to understanding the neurocognitive variables that influence learning, as their characteristics support effective data collection in natural environments where teaching and learning processes occur (Gashaj et al., 2024).

One such environment recently explored with EEG instruments is Virtual Reality (VR), an innovative space where students can engage in different activities. In 2021, a study compared two groups of students taking a reading test inside and outside of virtual reality. It found that the VR-reading condition showed higher theta band frequency activation at the parietal electrode sites (P3, P4, POZ), with a significant difference noted at the POZ electrode. In contrast, the alpha frequency band exhibited consistently lower activation across all electrode locations in the VR-reading condition compared to the Real-reading condition (Baceviciute et al., 2021). Another study demonstrated the possibility of distinguishing various levels of mental workload by analyzing electrical activity recorded from the scalp during an interactive VR task (Tremmel et al., 2019).

Additionally, a recent study guide for a group of educational researchers explored neural dynamics in the context of more complex, naturalistic stimuli: algebraic (symbolic) and geometric (non-symbolic) proofs. Students found geometric proofs more challenging to understand than algebraic ones, which contradicts the assumption about the intuitive appeal of visual, non-symbolic reasoning (Gashaj et al., 2024). However, students rated algebraic proofs as more familiar. The exploration of neural oscillations during the processing of geometric and algebraic proofs revealed distinctive patterns. Parietal electrodes showed greater activation than frontal ones during both the extended presentation of proofs and the first 200 ms, supporting the involvement of the frontoparietal network in mathematical processing (Gashaj et al., 2024). This illustrates the crucial role EEG tools play in understanding students' reasoning on specific topics.

Finally, another emerging topic in the field of education involves investigations into how different technologies impact learning processes. These studies are being conducted not only in Higher Education but also in industrial learning scenarios, focusing on interactions with technologies such as metaverse (Tlili et al., 2022), robots (Tan et al., 2019), and augmented reality (Villanueva et al., 2022). This growing interest has led to an increased focus on measuring cognitive and emotional processes using EEG instruments while students interact with machines (Casamadrid et al., 2024).

## 3 Materials and methods

This section describes the process of collecting, developing, and analyzing data related to this work. It includes subsections that detail participant selection criteria, demographic characteristics, neuropsychological evaluation to evaluate cognitive functions, classifying participants' performance according to age and educational level. In addition, EEG data collection performed with the use of Emotive Insight 2.0 headset during neuropsychological assessment tasks, recording biometric performance metrics. Along with this, the Procedure subsection describes the methodology of the data collection sessions. Overall, this section provides a comprehensive overview of the study methodology, crucial for understanding human skills in the context of Industry 5.0.

### 3.1 Participants

The EEG and *Neuropsi* test data were obtained from a group of 15 student volunteers. All students in this sample were from the engineering school of Tecnologico de Monterrey (average age of 20.7 years, SD = 2.5). The students belonged to the following programs: six fourth semester students from B.S Innovation and Development Engineering, three students from B.S in Sustainable Development Engineering in the fourth and sixth semesters, two students from B.S Mechatronics Engineering in the eighth and fourth semesters, two fourth semester students from B.S in Industrial and Systems Engineering, a second-semester student from B.S. in Civil Engineering and an eighth-semester student from B.S. in Computer Science and Technology. These participants had no recorded background of neurological disorders. All subjects provided consent after being adequately informed.

### 3.2 Neuropsychological assessment

As we mention in the introduction, the arrival of industry 5.0 implies an interdisciplinary approach in the search for new methodologies in fields such as industry and education. Within the framework of this interdisciplinarity, the use of biometric instruments and tests developed from areas such as Neuropsychology respond to a methodological novelty framed in the needs of industry 5.0.

Neuropsi test is one of the most widely used instruments for neuropsychological screening in Latin American countries (Santos et al., 2020; Morlett Paredes et al., 2021), particularly in Mexico, Argentina and Peru, where it is standardized (Ostrosky-Solís et al., 1999; Querejeta et al., 2012; Marreros-Tananta and Guerrero-Alcedo, 2022). As the most used test in clinical settings for measuring various cognitive abilities, it offers statistical reliability due to its standardization across different age groups. The characteristics of this test enabled us to select it as a sufficiently sensitive tool for measuring certain students cognitive abilities, allowing us to link these abilities with biometric values.

A brief Neuropsychological Battery in Spanish to collect information on the students' abilities (Neuropsi breve) was administered (Ostrosky-Solís et al., 2019). This test evaluates a wide spectrum of cognitive functions including orientation (time, person, and space), attention and activation, memory, language (oral and written), visual-spatial and visual-perceptual aspects, and executive functions (see Table 1). The evaluation of these areas includes techniques that reflect the specific characteristics of each function and incorporates recent findings from neuroanatomical, cognitive neuropsychology, and neurolinguistics research.

**Table 1:** Test areas.

**Orientation**	**Attention and concentration**	**Memory- encoding**	**Memory- retrieving**	**Language**	**Reading**	**Writing**	**Executive functions**
Time	Digits	Words	Spontaneous	Denomination	Reading	Dictation	Similarities
Place	Visual detection	Semi-complex Figure	By categories	Repetition	x	Write out	Calculation
Person	20 - 3	x	Recognition	Comprehension	x	x	Sequencing
x	x	x	Semi-complex Figure	Semantic Verbal fluency	x	x	x
x	x	x	x	Phonological Verbal fluency	x	x	x

Neuropsi test has updated standardization data corresponding to 2018, where it was administered to 2,000 normal subjects between 16 and 85 years old. According to age, the sample was divided into four groups: 16–30, 31–50, 51–65, and 66–85. Owing to this standardization, this test features stratification according to four educational levels: 0 years of study, low 1–4 years of study, medium 5–9 years of study, and high 10–24 years of schooling. It is important to note that these educational levels include elementary education, as the test is also designed to evaluate cognitive processes in individuals with no formal schooling. Considering the level of education and age of the subject, the subject's performance can be classified as high standard, normal, mild-moderate alteration, and severe alteration. In accordance with the aforementioned characteristics of the Neuropsi test, our sample of students in this research was classified in the age group of 16–30 years old, and their educational level was high between 10–24 years. Also, their performance was found between normal and high normal categories.

**Figure 1:**
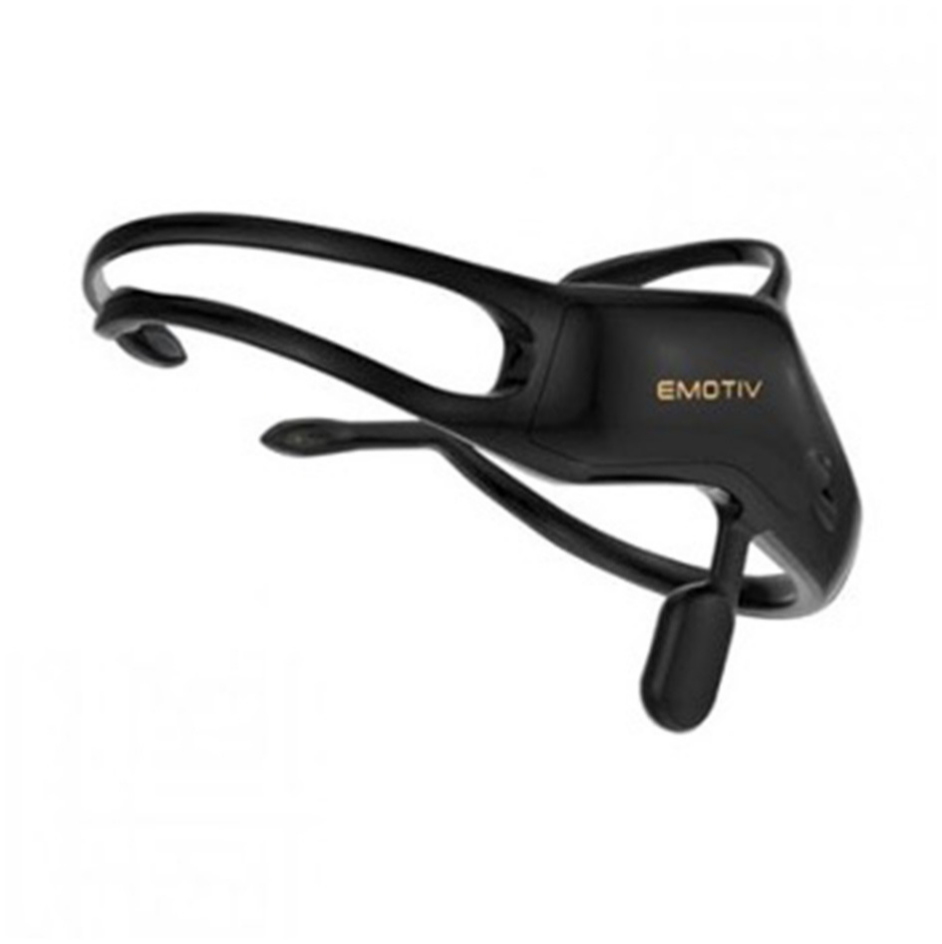
Emotiv Insight 2.0.

On the other hand, the EEG data was collected with the Emotive Insight 2.0 headset while the engineering students performed the Neuropsi tasks. Emotive Insight headset is an EEG system featuring five channels (as depicted in Figure 1) and utilizes semi-dry polymer sensors, functioning as a brain-computer interface (BCI). The EmotivPRO app enables recording performance metrics derived from the EEG data.

These metrics provide values for Engagement, Excitement, Attention, Interest, Relaxation and Stress. Performance Metrics are considered a biometric measure of cognitive and emotional aspects. This research considered four main performance metrics or mental states: Excitement, attention, engagement, and stress, as explained below.

- Stress: It measures the level of discomfort related to the task being performed.
- Engagement: It is related to a state of alert and conscious direction of attention toward the main stimuli to perform the task.
- Attention: It denotes a consistent focus on a singular task.
- Excitement: It is a sensation or perception of physical arousal accompanied by a favorable value.

### 3.3 Procedure

This study involved compiling the data from the Neuropsi test and the Performance Metrics to understand how human abilities could be measured. To accomplish this, we collected a group of engineering students to create a first evaluation protocol design. This data was collected by a student-by-student session that lasted around 1 h and 15 min each. At the beginning of each session, the student received a concise overview of the methodology and equipment. Later, the Emotiv Insight 2.0 device was positioned on the student's head, looking for the sensors to have good contact. Afterwards, a concentration strategy was sought to increase the quality of brain waves, and each student was helped to find the strategy that best suited them. Finally, when the connectivity quality was between 70 and 90%, the test application could begin with the EEG recording. This last part lasted about 25 min.

## 4 Results

The outcomes were obtained by testing 15 engineering students on their cognitive abilities. Also, EEG was collected while students performed the test to have a biometric counterpart. Regarding the Neuropsi test, a sample profile was summarized, showing that 60% of the students evaluated had a performance rating of High Normal, and the remaining 40% obtained a performance rating of Normal. None of the participants received a performance rating of mild-moderate or severe impairment. This shows that this test can classify engineering students into two profiles according to their performance in cognitive abilities.

On the other hand, there was the number of errors that the students made in the test (according to the Neuropsi test, the errors were counted due to omissions or incorrect answers within each of the areas of the test). Original score (N-score), standard score (Z-score), and the number of errors that each student obtained is shown in Table 2.

**Table 2:** Neuropsi test results.

**Student number**	**N-score**	**Z-score**	**Performance ratings**	**Errors**
S1	116	111	Normal	14
S2	123	120	High normal	7
S3	118	114	Normal	12
S4	119	115	Normal	11
S5	122	119	High normal	8
S6	118	114	Normal	12
S7	121	118	High normal	9
S8	122	119	High normal	8
S9	115	110	Normal	15
S10	128	127	High normal	2
S11	124	122	High normal	6
S12	127	126	High normal	3
S13	116	111	Normal	14
S14	127	126	High normal	3
S15	121	118	High normal	9

**Figure 2:**
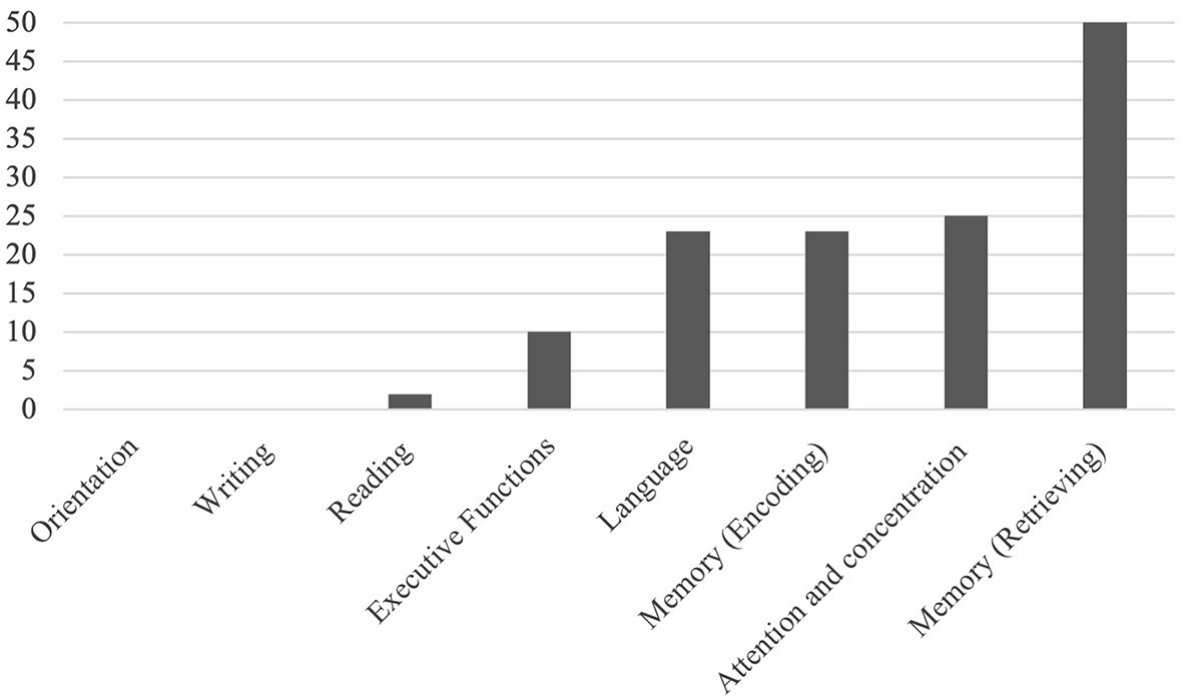
Number of errors in each of the Neuropsi test areas.

Errors indicated that the test areas for this sample of students were sensitive to measure errors. The area where students made the most errors was the Memory-retrieving area, with 50 errors in total, followed by the Attention and concentration area, with 25 errors, and the Memory-encoding and Language areas, with 23 errors each (see Figure 2).

In the areas of Writing, Reading, and Orientation, the students made almost no errors, which means that these areas are made with a different sensitivity because they are more aimed at measuring neurocognitive damage. This could suggest that for this group of engineering students, a plan could be developed to improve their Memory-encoding and retrieving, Attention and Concentration, and Language abilities to generate tools in the future workforce that help them be prepared for continuous changes that bring industry 4.0 and 5.0.

Related to the Performance Metric, EEG data showed the biometric values for four mental states: Attention, Engagement, Excitement, and Stress. The results were calculated by weighting the averages of the biometric values obtained by each student in the different test areas. According to this, it is possible to observe how each biometric result differs depending on the test area evaluated.

**Figure 3:**
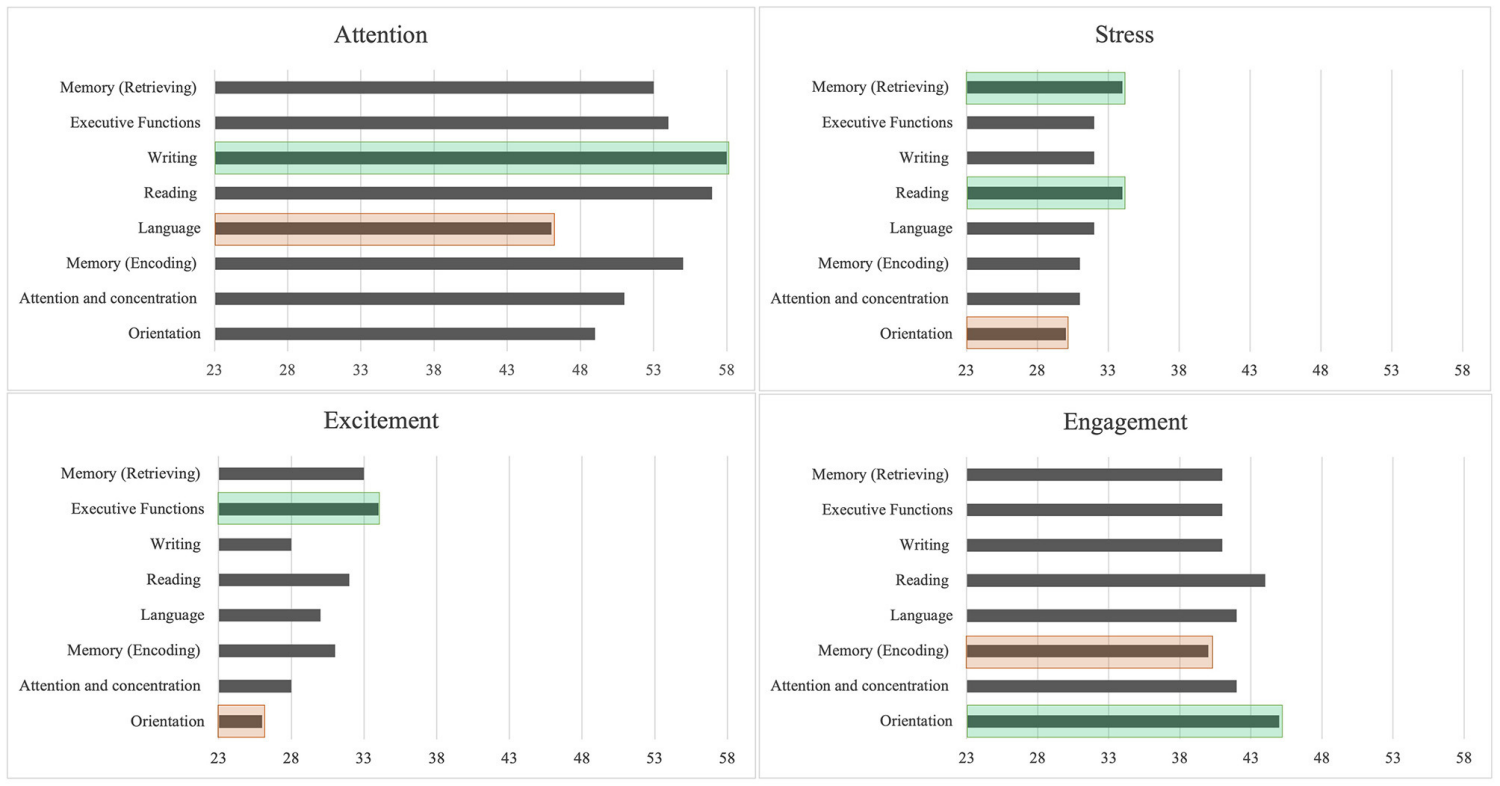
Mental states during Neuropsi test application.

The mental state with the highest biometric scores in all areas was Attention (see Figure 3). This shows how most students used brain waves associated with the cognitive process of attention to execute the different areas of the test.

Furthermore, the area that reported the highest biometric score in Attention was Writing, and the area that reported the lowest score in this mental state was Language, indicating which areas of the test consume more Attention than others in the group of engineering students evaluated.

The second mental state to obtain high scores was Engagement. Within this mental state, the area that received the highest score was Orientation, and the area that received the lowest score was Memory (Encoding). It is assumed that the moment of most significant student Engagement occurs in Orientation because it is the initial area of the test. For this reason, it is essential to observe how, after the orientation area, Engagement modulates according to the subsequent areas.

With these results, we obtained the lowest biometric values: Excitement and Stress. Related to Excitement, the area that obtained the highest biometric value was Executive Functions, and the area that obtained the lowest score was Orientation. As we explained before, excitement is the combination of physical arousal and the perception of favorable value toward something. This definition could be pointed out as to how areas that contain more complex and diverse sub-tests tend to produce more excitement in students.

Regarding Stress, the areas that obtained the highest scores were Memory (Retrieving) and Reading, and the area that obtained the lowest score was Orientation. It is also important to mention that the orientation area was one of the most minor challenging tests, which is why the evaluated students obtained zero errors in their performance (see Figure 2). Furthermore, this could also explain why, in the biometric values, this area received the lowest score in Stress.

## 5 Discussion

Industry transitions present a paradigm shift in the industrial landscape, emphasizing human focus, collaboration, and innovation. As Industry 5.0 develops, it becomes imperative to address changing educational needs and workforce requirements, particularly in engineering education and creating knowledge, skills, and abilities (KSA)-based taxonomies. The assessment and categorization of KSA are crucial for developing effective training and educational programs in the context of Industry 5.0. Existing taxonomies, such as those developed by organizations like the World Economic Forum and the Government of Singapore's SkillsFuture initiative, provide comprehensive frameworks for understanding the essential competencies required for various occupations (SkillsFuture Singapore, 2021; WEF, 2021). These frameworks highlight the need for continuous skills development and lifelong learning to adapt to the rapidly evolving job landscape. Additionally, dynamic KSA taxonomies, such as the ShapingSkills framework, leverage systematic literature reviews, industry surveys, and emerging trends in the labor market to provide real-time insights into skill requirements, that make it incorporate advanced techniques like Natural Language Processing (NLP) and Artificial Intelligence (AI), these dynamic taxonomies offer a more responsive and up-to-date categorization of skills, addressing the limitations of static taxonomies (ShapingSkills, 2023). This approach aligns with the broader goals of Industry 5.0, which emphasize human-centered innovation and the integration of new technologies into the workforce (Redecker, 2017; Breque et al., 2021; OECD, 2023). Integrating neurobiological insights with these taxonomies could further enhance our understanding of cognitive abilities and how they relate to job performance, ultimately leading to more effective training programs and better-prepared professionals for the future workforce (Fragkaki et al., 2022).

Having this industry transition latent, a redefinition of the CEE is necessary to encompass the integration of sustainability, resilience, and human-centered principles in upskilling and reskilling initiatives (Grodek-Szostak et al., 2020; Breque et al., 2021). While Industry 4.0 laid the foundation for digitalization and AI-driven technologies, Industry 5.0 emphasizes social objectives, environmental considerations, and employee wellbeing (Xu et al., 2021). As such, CEE programs should evolve to address these changing priorities, preparing engineers to navigate the complexities of Industry 5.0 while promoting lifelong learning and professional development (Cuckov et al., 2022; Lantada, 2022).

The assessment of S&A is an essential element both in universities and Industries. Companies employ people closest to the Skills and Abilities profile required by each job position evaluating them with different instruments. For this reason, Industry 5.0 places humans at the forefront of technological innovation, highlighting the importance of understanding and evaluating the skills and abilities of engineering graduates (Breque et al., 2021; Troisi et al., 2023).

This methodology, which combines a cognitive abilities test with a biometric, is the first interdisciplinary approach to recognize from different perspectives how the abilities of engineering students could be characterized. As a result of this combination, it was possible to observe that the Neuropsi test is an instrument that could evaluate some cognitive abilities of the engineering students classified as normal and high normal, especially those areas of the test where the students showed variations in the number of errors such as Language, Memory-encoding, Memory-retrieving, and Attention and concentration. Other authors have used the Neuropsi test with different approaches, for instance, to measure cognitive abilities such as attention and memory in students with low reading comprehension (Pérez et al., 2020). Also, this test was tested searching for a Tele-neuropsychological adaptation due to the increase in the necessity to perform virtual neuropsychological evaluations during the COVID-19 period (González-Osornio et al., 2022).

One of the main objectives of HE is to prepare qualified professionals who nourish the workforce. However, the rapid pace of technological advancement contributes to a skills gap and mismatch within the engineering workforce (Schwab, 2017; WEF, 2018). Therefore, HE institutions should search for resources that help them understand the development of cognitive abilities in students. CN is one of those new approaches that has brought techniques and methodologies to different fields. For instance, EEG and BCI techniques have been used in the education field to understand how Mathematical Mindset theory should increase student motivation (Daly et al., 2019). With these same techniques, it has also been possible to understand the students attention process while they are in an online class (Al-Nafjan and Aldayel, 2022).

As depicted above, implementing biometric technologies allows us to delve deeper into not only the cognitive but also emotional aspects of students (Rajendran et al., 2022). According to this, our biometric data obtained from EEG allowed us to recognize the level of four mental states of the 15 engineering students while they performed the Neuropsi test. Two of these states were cognitive: Attention and Engagement. And the other two mental states were emotional: Stress and Excitement. It is interesting to observe how cognitive mental states had higher scores than emotional states, but even though the Neuropsi test measures cognitive abilities, stress and excitement were present in all students. Furthermore, these two emotional states fluctuated in the different test areas.

Along with this, it can be indicated that neurobiological studies offer a promising way to evaluate the cognitive abilities of engineering students in the context of Industry 5.0 (Aguayo-González et al., 2021). Researchers can gain valuable insights into students' cognitive strengths, learning preferences, and emotional regulation abilities (Apicella et al., 2022; Pathak and Kashyap, 2022) by leveraging EEG data collection and neuropsychological assessments. This knowledge allows the development of specific training programs adapted to individual learning styles and cognitive strategies, facilitating successful integration into the workforce (Nandi et al., 2021; Apicella et al., 2022). Additionally, neuroeducation approaches can help identify and develop soft skills increasingly valued in Industry 5.0 roles (Jolles and Jolles, 2021).

The design of collaboration spaces can also benefit from this neurobiological approach. In terms of hybrid collaboration spaces using immersive technologies, a Human-Centered approach can be used for the design of an environment where employees can feel comfortable (Mimnaugh et al., 2023) and participative (Kee et al., 2023) to improve their performance.

Integrating neurobiological approaches into engineering education represents a significant step in addressing the skills requirements of Industry 5.0. Through this work, it can be indicated that researchers could obtain a comprehensive understanding of student's cognitive abilities and adapt educational interventions by seeking to combine neuropsychological assessments with EEG data collection. This considers that these evaluation methods must be further refined, their applicability explored more broadly in different engineering disciplines, and their long-term impact on workforce preparation and performance investigated.

All this taking into account that Industry 5.0 continues to reshape the industrial landscape, and it becomes essential to adopt innovative approaches to be applied in education and skills assessment. So, by leveraging neurobiological insights and dynamic taxonomies, educators and industry stakeholders can ensure that engineering graduates are equipped with the diverse set of skills potentially needed to thrive in the era of Industry 5.0.

Finally, it is important to mention that there is ample information about the ethical concerns and correct uses of biometrics in areas such as identity (Sutrop and Laas-Mikko, 2012) and privacy (Tanwar et al., 2019). However, in the specific area of educational research, these concerns still remain. With the increased use of biometrics such as EEG, it is crucial to guide efforts in this direction to enrich neuroeducation studies with ethical and safe protocols for students and teachers, similar to the standards established for VR environments (Christopoulos et al., 2021).

## 6 Conclusions and future work

This work shows a combined methodology that allowed us to have a first approach to the cognitive abilities of engineering students from neurobiological and behavioral perspectives. Highlighting the abilities and skills assessment as a transversal topic within industry 5.0 and CEE frameworks. Additionally, two profiles were extracted from the results. The first profile illustrates the Neuropsi test as an instrument that could evaluate some cognitive abilities in engineering students since it shows the tendency of this sample to have more errors in some cognitive areas than others. The second profile presents the EEG and Neuropsi test interaction, showing engineering students' cognitive and emotional states based on biometric levels associated with the test areas. This indicates the relevance of using biometric measurement tools to have more complete student profiles.

As we said previously, this is the first approach to evaluating cognitive abilities in engineering students. However, to achieve the definition of an evaluation protocol that adapts to our KSA taxonomy, it is necessary to continue exploring other tests and assessment tools to continue integrating the pull of abilities framed in Industry 5.0.

In the future, one of the benefits that the design of an evaluation protocol can bring is integrating its S&A diagnoses into training programs and human-centered collaboration spaces.

## Data Availability

The raw data supporting the conclusions of this article will be made available by the authors, without undue reservation.
